# Graph Neural Networks for Parkinson’s Disease Monitoring and Alerting

**DOI:** 10.3390/s23218936

**Published:** 2023-11-02

**Authors:** Nikolaos Zafeiropoulos, Pavlos Bitilis, George E. Tsekouras, Konstantinos Kotis

**Affiliations:** Intelligent Systems Laboratory, Department of Cultural Technology and Communication, University of the Aegean, 81100 Mytilene, Greece; cti22009@aegean.gr (N.Z.); pavlos.bitilis@aegean.gr (P.B.); gtsek@ct.aegean.gr (G.E.T.)

**Keywords:** knowledge graphs, graph neural networks, Parkinson’s disease

## Abstract

Graph neural networks (GNNs) have been increasingly employed in the field of Parkinson’s disease (PD) research. The use of GNNs provides a promising approach to address the complex relationship between various clinical and non-clinical factors that contribute to the progression of PD. This review paper aims to provide a comprehensive overview of the state-of-the-art research that is using GNNs for PD. It presents PD and the motivation behind using GNNs in this field. Background knowledge on the topic is also presented. Our research methodology is based on PRISMA, presenting a comprehensive overview of the current solutions using GNNs for PD, including the various types of GNNs employed and the results obtained. In addition, we discuss open issues and challenges that highlight the limitations of current GNN-based approaches and identify potential paths for future research. Finally, a new approach proposed in this paper presents the integration of new tasks for the engineering of GNNs for PD monitoring and alert solutions.

## 1. Introduction

Categorized as a progressive neurodegenerative disease, PD exerts its impact on millions of individuals globally. Despite extensive scholarly inquiry, the precise etiology of PD remains largely elusive, and the currently available therapeutic regimens exhibit constrained effectiveness in retarding or arresting the disease’s relentless progression. Recent times have witnessed a burgeoning emphasis on the utilization of data-driven methodologies, specifically machine learning (ML) and deep learning (DL) ones, as instruments for probing into the fundamental mechanisms underpinning PD and augmenting the efficacy of therapeutic interventions.

Neural networks (NNs), inspired by the human brain, learn patterns from data and are widely used in healthcare for tasks like disease diagnosis, drug discovery, and personalized treatment planning [1]. In PD research, they constitute a promising technology in predicting progression, categorizing stages, and detecting early signs [2]. However, NNs have limitations, including their ‘black box’ nature, big data demands, risks of overfitting or underfitting, and computational intensity, which can hinder interpretation, explainability, and generalization.

In contrast to traditional NNs, GNNs are particularly well-suited for graph-structured data, characterized by varying sizes and complexity of structure. GNNs excel at capturing intricate relationships within graphs, making them highly effective in tasks such as node classification, link prediction, and graph classification [3]. As a specialized form of DL, GNNs are designed explicitly for graph-structured data, commonly found in domains like social networks and molecular biology [4]. Their strength lies in their ability to incorporate both local and global structural information for more accurate predictions, enabling them to uncover intricate relationships among entities.

Compared to traditional NNs, GNNs are preferred for the representation of graph-structured data. While NNs are designed for vector or sequence data, GNNs excel at analyzing complex graphs by leveraging node and edge relationships. They are particularly useful in domains with inherent graph-based data, such as traffic analysis, social networks, and recommendation systems [5]. In medical applications, including the PD domain, GNNs play a pivotal role in tasks like gene expression analysis, disease diagnosis, drug discovery, and brain imaging data analysis. Their graph-centric approach serves as a robust tool for the analysis of complex medical data, yielding novel insights and interpretations of disease diagnosis and treatment. In the context of neurodegenerative disorders like PD, a representative recent study introduced a GNN-based method to forecast PD progression by analyzing brain connectivity networks from MRI data [6]. Notably, this GNN model can identify changes in brain connectivity patterns that are predictive of disease progression, even in the early stage of PD, offering a promising approach for early diagnosis and improved treatment strategies [7]. Additionally, in another related work [8], PD was recognized as a common neurodegenerative ailment and a widespread condition influenced by a blend of genetic and environmental factors, contributing to the formation of abnormal protein aggregations in specific neuron groups, ultimately resulting in cellular dysfunction and degeneration. The clinical diagnosis of PD often relies on a careful evaluation to distinguish it from other parkinsonism-related disorders, necessitating a heightened level of clinical suspicion. A multitude of treatment modalities, including pharmaceutical agents and surgical interventions, are now available to address both early- and late-stage complications associated with PD.

Statistical reasoning in healthcare, especially in PD research, involves data-driven inferences, aiding in risk identification, intervention assessment, and uncovering progression patterns. Symbolic reasoning in PD healthcare research uses symbols for structured problem-solving, contributing to the understanding of complex relationships among symptoms, biomarkers, and treatments, enabling personalized treatment planning. It also extracts information from patient records to cover disease progression and treatment response.

Hybrid AI combines statistical and symbolic approaches, blending data-driven and knowledge-driven methods [9]. This blending addresses complex problems by leveraging the strengths of both approaches [10]. For example, probabilistic soft logic (PSL) unifies statistical and symbolic reasoning in social network analysis and natural language processing, and inductive logic programming (ILP) extracts symbolic rules from data for application in bioinformatics and expert systems [11].

The key contributions of our work are:

- Emphasize the integration of cutting-edge technologies, particularly GNNs, for the development of innovative solutions in PD research.
- Provide a comprehensive overview of GNNs in PD research, including open issues and challenges, and a proposed approach to tackle them.
- Highlight the need for the semantic representation and integration of sensor/historic data from disparate and heterogeneous sources, such as wearable technology and electronic healthcare records (EHRs), into GNN frameworks.
- Introduce a novel comprehensive and cohesive approach to harness the full potential of GNNs in PD research.
- Present an innovative edge-device-based application for real-time health data collection, integration, and analysis for generating timely alerts for missed medication doses.
- Assist experts/scientists in bridging the gap in understanding the full scope of GNN applications in PD and their potential impact on disease management.
- Highlight opportunities that GNNs bring to PD research for a deeper understanding of the disease, paving the way for more effective treatments and personalized care.

The structure of this paper is as follows: Section 2 provides essential background knowledge on the subject. Section 3 outlines the research methodology adopted for this survey. In Section 4, we present a curated selection of state-of-the-art scientific studies. Section 5 discusses open issues and challenges for expanding the role of GNNs in the domain of personal health monitoring for PD. Moving beyond the state-of-the-art, Section 6 introduces a proposed framework for GNN use in PD patient care. Finally, Section 7 concludes the paper and outlines plans for future research.

## 2. Background Knowledge

In this section, our objective is to offer a comprehensive overview of fundamental concepts and prior scientific investigations in the domain of utilizing GNNs for PD. This review serves the dual purpose of establishing a knowledge baseline and identifying the most promising avenues for the development of a reference architecture for GNNs in PD treatment and prevention [12]. It encompasses the elucidation of key terminologies and a meticulous examination of pertinent scientific literature [13]. The concepts to be addressed are outlined in the following paragraphs.

Data analysis assumes a pivotal role in contemporary medical research, with relevance in the context of PD [14]. PD stands as a complex and progressive neurodegenerative disorder, marked by a spectrum of symptoms encompassing tremors, rigidity, and motor impairments. To fathom the underlying mechanisms of PD necessitates the analysis of extensive and diverse datasets, including clinical and non-clinical data drawn from various sources [15]. These sources encompass EHRs, patient-reported outcomes, and imaging data.

In the landscape of PD research, data analysis has traditionally relied upon classical statistical methodologies such as regression analysis and hypothesis testing. However, the burgeoning availability of high-dimensional data and the increasing complexity of relationships between variables necessitate the adoption of more advanced techniques [16]. This shift has ushered in the prominence of ML and DL methodologies as formidable instruments for dissecting intricate datasets, unveiling latent patterns, and discerning intricate relationships.

In the context of PD research, ML and DL techniques prove invaluable, enabling the identification of pertinent features, the stratification of patients based on the severity of their symptoms, and the prediction of disease progression [17]. Additionally, these techniques provide the means to explore the intricate associations between various clinical and non-clinical factors, encompassing demographic parameters, genetics, lifestyle choices, and environmental influences, and their collective impact on the trajectory of PD [18]. Consequently, data analysis assumes a pivotal role within the domain of PD research, holding the potential to not only deepen our comprehension of the ailment but also to foster the development of more efficacious treatments. The integration of advanced ML and DL methodologies within this field promises to yield fresh insights into the intricate underpinnings of PD, ultimately enhancing outcomes for patients. In [19], the authors highlight the diagnostic challenges in identifying PD due to the absence of well-defined clinical markers, emphasizing the importance of nonmotor symptoms as significant contributors to patient disability and suggesting their occurrence even before the motor symptoms. The review discusses the evolution of PD diagnosis criteria, with particular focus on the incorporation of nonmotor symptoms in current guidelines, aiming to improve diagnostic accuracy, especially in the early stages. The use of molecular markers and smart devices is suggested as potential strategies for detecting prodromal PD symptoms, enabling early disease identification and the development of novel neuroprotective therapies.

In PD research, data preparation plays a pivotal role. Datasets from sources like EHRs, patient-reported outcomes, and imaging data often possess diverse formats, structures, and varying data quality [20]. Data preparation encompasses crucial tasks such as cleaning, integration, normalization, and transformation, ensuring data suitability for rigorous analysis while accurately reflecting variable relationships. PD research, marked by data complexity and heterogeneity [21], heightens the importance of data preparation. For example, EHRs may contain missing or inconsistent data, patient-reported outcomes use varying measurement scales, and imaging data exhibit resolution and orientation variations. Thus, meticulous data preparation becomes pivotal, ensuring result accuracy and reliability. Indeed, data preparation is an indispensable component of PD research, influencing result quality [22]. Effective data preparation techniques overcome challenges posed by data intricacies, facilitating deeper insights into PD’s underlying mechanisms.

EDA assumes a pivotal role in PD research, primarily due to the disorder’s complexity and the diverse data sources it encompasses [23]. EDA serves as a critical means of discerning data patterns, relationships, and anomalies, thereby facilitating a comprehensive grasp of the data’s underlying structure. This holds particular significance in the context of PD research, where the intricacies and heterogeneity of data necessitate the discovery of concealed patterns and relationships that can profoundly impact disease progression [24].

In the domain of PD research, EDA employs an array of visualization techniques and descriptive statistics, encompassing scatter plots, histograms, and heat maps, as well as metrics like mean, median, and standard deviation. These analytical tools are instrumental in unearthing trends such as the temporal distribution of symptoms, associations between demographic variables and disease evolution, and the influence of lifestyle and environmental factors on PD [25]. Effectively conducted EDA contributes significantly to a comprehensive understanding of data and their inherent structure. By unveiling hidden data patterns and relationships, EDA harbors the potential to furnish fresh insights into the fundamental mechanisms of PD, ultimately guiding the development of more efficacious treatments.

In continuation of the analytical progression outlined in the preceding stages, a knowledge graph emerges as a graph-based model that organizes information into a structured format, employing nodes and edges. Within the framework of PD, a knowledge graph finds utility in representing relationships among various entities, encompassing genes, proteins, drugs, and diseases, while capturing the scientific knowledge pertaining to PD from the literature. Its functionality extends to reasoning over this knowledge, thereby enabling the inference of novel relationships among entities. For instance, a knowledge graph can facilitate the prediction of potential drug targets for PD based on established interactions among genes, proteins, and drugs. On a parallel note, GNNs, a specialized category of NNs, operate directly on graphs. They are meticulously designed to acquire node and edge representations within a graph, effectively encapsulating the intrinsic relationships that bind them. In the context of PD, GNNs prove instrumental in tasks such as classifying PD patients [26] based on their symptoms, predicting disease progression, or identifying potential biomarkers of the ailment. Their utility is particularly pronounced when dealing with data presented in a graph format, as is often the case with brain imaging data or social network data. In [27], the authors delve into the contributions and applications of the Internet of Things (IoT) and the Internet of Medical Things (IoMT), with a specific focus on dementia patient care and early detection. The study highlights the extensive use of sensors and smart devices for remote monitoring of patients’ vital statistics, especially those with dementia. It emphasizes the potential of smart IoMT-enabled systems in early dementia detection, offering the opportunity for better disease management and enhanced patient quality of life. The proposed model “Demencare” leverages IoT and edge computing to provide 24/7 patient monitoring, addressing the challenges in dementia care. The incorporation of fog computing is suggested to further enhance the capabilities of IoMT, promising improved patient care and diagnostic outcomes.

In the context of PD, a knowledge graph serves as a repository encompassing diverse facets of information pertinent to the ailment. This wealth of data spans aspects including symptoms, etiological factors, risk determinants, therapeutic modalities, and clinical trial data. Within this kind of graph, intricate relationships interlink various concepts, elucidating connections such as the correlation between specific genes and PD onset, the influence of environmental variables on disease progression, and the efficacy of different medications in symptom management. To illustrate, a knowledge graph can unveil the intricate involvement of particular genes in PD pathogenesis while delineating their interactions with factors like age and environmental toxins [28]. Additionally, it can portray the interplay between distinct PD symptoms, such as tremors and rigidity, and the array of available treatment options aimed at symptom alleviation.

The current approach to monitoring and alerting for pill dosing in PD relies on fixed-time alerts on smart devices, which are based on daily dosage interval (DDI) values entered by the patient or their doctor in the configuration settings of the app. However, this approach does not take into account the real-time health status of the patient, such as their immediate symptoms or condition, and solely relies on the patient’s actions, potentially leading to deviations from the treatment plan. This lack of real-time health data integration can result in suboptimal treatment adherence and effectiveness. DDIs refer to the specific times at which medications or treatments are scheduled to be administered or taken each day. They define the intervals or time gaps between doses of a particular medication or therapy within a 24 h period. DDIs are commonly used in healthcare to ensure that patients receive their medications at the prescribed times and in the correct doses, which is crucial for effective treatment, especially in conditions like PD where medication adherence is important for symptom management.

**Figure 1:**
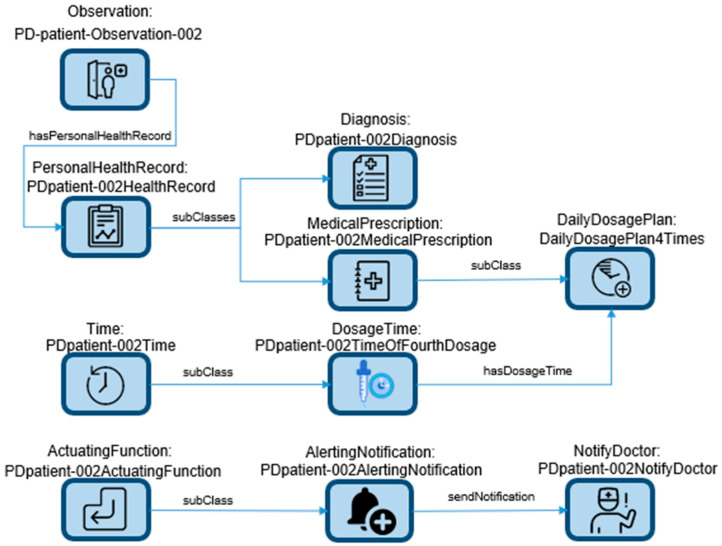
Graph representation of a PD monitoring scenario for recognizing a missing dose event.

GNNs represent a class of algorithms ideally suited for handling graph-structured data, a prevalent data format in PD research [29]. These algorithms leverage graph-based data representation to effectively model relationships between variables, allowing for predictive modeling and pattern recognition. The process of constructing a knowledge graph involves scrutinizing the data within a knowledge base to extract triples, followed by their insertion based on a graphical data model. This integration of data triples results in the formation of a comprehensive knowledge graph, encompassing five distinct entity types: genes, diseases, drugs, channels, and side effects. Notably, these entities are interconnected, fostering a web of interrelationships among them. A partial example representation of a data schema is shown in Figure 1. The ontology data schema serves as the foundation for the development of a medication adherence monitoring and alert system tailored for PD patients. The system’s primary objective lies in facilitating timely medication intake while minimizing instances of missed doses. Additionally, it incorporates mechanisms to promptly notify both the patient and their healthcare provider in the event of emergency situations.

The depicted scenario describes a practical application within the realm of PD. The existing approach to monitoring and alerting for pill dosing in PD primarily relies on fixed-time alerts through smartphones or tablets, which are contingent upon patient or healthcare provider inputs. This method does not take into consideration the patient’s actual health status, allowing the patient to confirm dosing at any time, irrespective of alignment with the prescribed treatment plan. To address this limitation, a novel approach is proposed, centered around an edge-device-based application [30]. This application functions by continuously collecting and analyzing real-time or near-real-time personal health data from PD patients. It aims to discern instances of missed doses by examining low-level events such as bradykinesia (slowness of movement) and tremors [31]. This novel approach employs a knowledge-graph-based methodology to model relationships among various variables and symptom patterns, thus facilitating accurate predictions and alerting either the patient or their healthcare provider about missed doses. Leveraging the knowledge graph framework enables the system to uncover previously unrecognized connections between different variables and symptom patterns, ultimately enhancing the precision of missed dose detection and optimizing the management of PD symptoms.

Conversely, a GNN possesses the capability to absorb data encompassing PD patient symptoms, medication dosages, and temporally tagged information regarding medication adherence. Subsequently, it constructs a graph-based representation that encapsulates the patient’s evolving health status over time. The GNN leverages message-passing algorithms to disseminate information throughout the graph, enabling the acquisition of insights into the relationships between diverse variables and symptom patterns. This acquired knowledge empowers the GNN to make precise predictions regarding the occurrence of missed dose events and to communicate timely alerts to either the patient or healthcare provider. In contrast to the conventional fixed-time alerts approach, the GNN methodology excels in terms of accuracy and effectiveness in the management of PD symptoms.

As depicted in Figure 1, the ontology data schema collectively holds the potential to enhance medication adherence and mitigate adverse events among PD patients. It achieves this by offering personalized and timely alerts rooted in the patient’s medication regimen and medical history.

This survey underlines the significance of data representation within the realm of GNNs for PD research. During the developmental phase, the utilization of data visualization emerges as a pivotal component, aiding in the augmentation of accuracy and robustness in GNN models. It accomplishes this by facilitating the recognition of inter-variable relationships and the detection of potential data anomalies [32]. Furthermore, data visualization assumes a crucial role in the interpretation of GNNs. It empowers researchers to uncover data patterns and comprehend the intricate web of relationships among variables, thereby enabling fresh insights into the underlying mechanisms of PD and contributing to the refinement of novel treatment strategies [33].

ML is a swiftly advancing field, holding substantial promise in the exploration of PD. PD, being a multifaceted and progressively deteriorating neurodegenerative condition, afflicts millions worldwide. A comprehensive understanding of its underlying mechanisms necessitates the scrutiny of extensive and diversified datasets [34]. These datasets originate from diverse channels, including EHRs, patient-reported outcomes, and imaging data, collectively harboring a reservoir of insights regarding the disease and its progression.

GNNs constitute a specialized category of data processing (DP) algorithms tailored for managing graph-structured data, commonly encountered in PD research datasets. By embracing graph-based data representation, these algorithms effectively model interconnections among variables, enabling both pattern recognition and prediction. Additionally, ML proves invaluable in PD research due to its capability to analyze extensive and intricate datasets, unveiling latent patterns and relationships that significantly influence disease progression [35]. In the context of PD research, ML is instrumental in constructing predictive models for estimating disease trajectories, identifying patient cohorts with similar profiles, and devising personalized treatment strategies. Together, ML and GNNs play pivotal roles in advancing PD research, offering a robust and versatile approach to comprehending the ailment’s underlying mechanisms and optimizing therapeutic strategies [36]. Through in-depth analysis of complex datasets and the revelation of hidden patterns and relationships, these technologies provide fresh insights into PD and serve as valuable resources for enhancing treatment modalities.

GNNs constitute a category of DL algorithms purposefully engineered to manage graph-structured data in the context of PD research, encompassing datasets such as patient-reported outcomes, imaging data, and EHRs [37]. Through their capacity to model inter-variable relationships within the data, GNNs have the potential to yield novel insights into the underlying mechanisms of PD and facilitate the development of more efficacious treatment modalities [32]. Concurrently, DL stands poised to provide substantial contributions to PD research by offering a potent and adaptable instrument for scrutinizing intricate datasets. This capability enables the unveiling of concealed patterns and relationships, thereby unearthing fresh perspectives on the progression of PD and streamlining the creation of individualized treatment regimens for patients.

In summary, the utilization of DL and GNNs stands as a fundamental requirement in PD research, providing a versatile and potent approach to comprehending the underlying mechanisms of the disease and devising efficacious treatments [38]. By meticulously scrutinizing intricate datasets and unveiling obscured patterns and interrelations, DL and GNNs possess the capacity to yield innovative perspectives on the disease and its progression. This, in turn, holds the potential to enhance the development of more efficient treatment strategies.

PD is a neurological disorder characterized by the degeneration of dopamine-producing neurons in the substantia nigra, resulting in a spectrum of motor and non-motor symptoms. The comprehension and management of this intricate ailment represent a substantial challenge in healthcare. NNs offer a promising avenue for shedding light on the underlying mechanisms of PD and the development of more effective therapeutic strategies. Through the modeling of intricate interconnections among various factors encompassing patient-reported outcomes, imaging data, and EHRs, NNs possess the potential to unveil concealed patterns and relationships that are pivotal in unraveling the complexities of PD progression. PD represents a neurodegenerative ailment characterized by the deterioration of dopaminergic neurons in the substantia nigra, resulting in a spectrum of motor and non-motor symptoms [35]. The intricate nature of PD, coupled with the multitude of factors influencing its advancement, presents a substantial hurdle in gaining a comprehensive understanding of the underlying disease mechanisms and in the pursuit of effective therapeutic interventions. GNNs hold the potential to catalyze advancements in PD research by offering a robust and adaptable tool for dissecting graph-structured data [39]. These algorithms can be trained to discern intricate inter-variable relationships and derive predictions based on these relationships. This has the potential to yield fresh insights into the fundamental mechanisms governing PD and contribute to the refinement of more efficacious treatment approaches.

NNs play a pivotal role in PD research as they provide a robust and flexible approach to analyzing intricate datasets, uncovering hidden patterns, and discerning correlations. By modeling the intricate connections between various factors, NNs hold the potential to reveal fresh insights into the fundamental mechanisms underlying PD, thereby aiding in the development of more effective therapeutic interventions. In recent years, GNNs have gained prominence due to their proficiency in capturing complex relationships within graph-structured data, with applications spanning various domains, including healthcare. Table 1 provides an overview of the technologies (machine learning, deep learning, data preparation, data visualization, GNNs, and NNs) utilized in the context of the related works.

## 3. Research Methodology

A comprehensive literature survey was conducted, utilizing Google Scholar as the primary search tool. The search strategy involved a combination of pertinent keywords, including: “Data Analysis”, “Data Preparation”, “Exploratory Data Analysis”, “Data Visualization”, “ML”, “DL”, “Graph NNs”, and “Electronic Healthcare Records”. These keywords were also systematically paired with “PD” to ensure comprehensive coverage. All types of scholarly works were considered eligible for inclusion in the review, encompassing conference proceedings, dissertations, book chapters, peer-reviewed archived manuscripts, and published peer-reviewed manuscripts.

The search queries outlined earlier yielded a total of 300 papers from Google Scholar and 40 papers from ResearchGate. A thorough manual review of these papers was conducted to identify those with a specific focus on the utilization or construction of GNNs within the healthcare domain, with PD being the primary subject of study. This meticulous review process led to a refined selection of 143 papers meeting the criteria. Additionally, during the phase of data collection, a total of 340 documents were gathered from various conferences. Subsequently, prior to initiating the article screening process, 18 duplicate records were identified and removed, while 28 records were deemed ineligible, and 60 documents were excluded for various reasons. As the screening phase commenced, 234 articles were initially considered. Of these, 41 were excluded, and an additional 17 reports were unobtainable. Consequently, following the conclusion of the article screening phase, 176 reports remained for further assessment of eligibility. Of these, certain documents were eliminated due to non-relevance to the research objectives after a thorough reading. Upon concluding the article screening phase, 143 sources remained, and notably, 94 of these represented articles that referenced an additional 45 sources, all of which contributed to the comprehensive bibliographic review.

**Figure 2:**
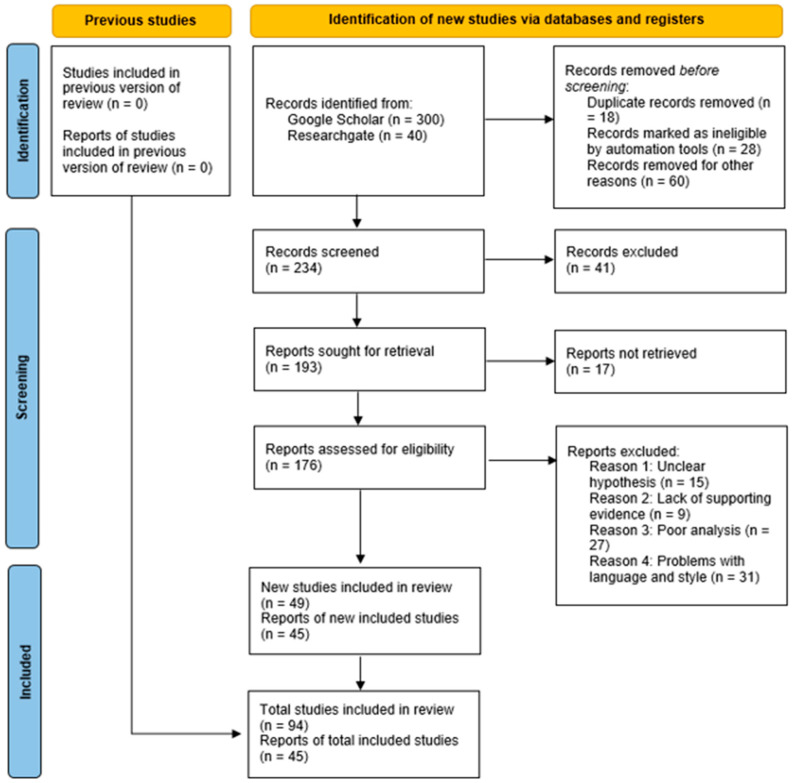
PRISMA research methodology.

Subsequently, this collection of papers underwent further refinement to exclusively encompass those papers that had been published or made available in public manuscript archives during the period spanning from January 2018 to December 2022, with their full-text versions publicly accessible at the time of composing this study. This meticulous process culminated in a final set of 94 papers. Additional details and insights are illustrated in Figure 2.

Figure 2 provides an overview of the paper selection process. By amalgamating the outcomes of our searches on ResearchGate and Google Scholar, we systematically applied the research methodology known as PRISMA. Initially, a rapid review was conducted to winnow down the initial pool of papers. Subsequently, each paper underwent meticulous scrutiny, coupled with additional techniques, leading to the ultimate inclusion of 75 papers in our study.

## 4. State-of-the-Art in GNNs for PD

The realm of GNNs in PD research is rapidly evolving, witnessing an upsurge in novel approaches. Recent years have witnessed growing interest in employing GNNs to explore PD’s underlying mechanisms, thanks to their robust data analysis capabilities. An inherent strength of GNNs in PD research lies in their adeptness at handling vast datasets from various sources, including patient-reported outcomes, imaging data, and EHRs. By integrating these datasets into a knowledge graph, GNNs can unveil intricate data patterns and relationships, providing insights into PD’s fundamental mechanisms. Furthermore, advancements have emerged in using GNNs for PD diagnosis, including the use of DP models to analyze imaging data and predict disease progression, showing promising results in PD detection and prognosis. These approaches hold the potential to improve patient well-being and reduce the healthcare burden of PD. In summary, GNNs in PD research continue to evolve, with new approaches frequently reported. This dynamic field offers promise for advancing our understanding of PD and enhancing patient outcomes, representing a promising area for future research.

### 4.1. Graph Convolutional Networks (GCNs)

Graph convolutional networks (GCNs) represent a specific category of GNNs tailored for the analysis and manipulation of graph-structured data. In the context of PD research, characterized by intricate relationships between diverse data sources like patient-reported outcomes, imaging data, and EHRs, GCNs emerge as a fitting choice. Within GCNs, the graph’s nodes serve as data points, while edges denote the connections between these points. Through the application of convolutional filters on these graph structures, GCNs efficiently process and scrutinize data, accounting for the interdependencies among different data points. This unique ability empowers GCNs to unveil intricate data patterns and relationships, thereby facilitating a deeper understanding of the fundamental mechanisms underlying PD.

A notable advantage of GCNs in PD research lies in their capacity to handle substantial volumes of data from varied sources, encompassing patient-reported outcomes, imaging data, and EHRs. This integration of data into a knowledge graph enables GCNs to unveil complex data patterns and relationships, offering valuable insights into the underlying aspects of PD. Recent advancements in applying GCNs to PD diagnosis have emerged, notably in deploying DL models to scrutinize imaging data and forecast disease progression. These approaches exhibit considerable promise, characterized by robust accuracy rates in detecting PD and predicting its progression. Such developments signify the potential to enhance patient outcomes and alleviate the strain that PD places on healthcare systems.

This research has introduced a method called multi-view graph convolutional network (MV-GCN) designed for predictive tasks related to PD. MV-GCN utilizes multiple brain graph inputs from diverse perspectives to enhance prediction accuracy [40]. Validation of the MV-GCN method is conducted using real-world data from the Parkinson’s Progression Markers Initiative (PPMI), which tracks disease progression in patients. The method’s effectiveness is assessed in predicting pairwise matching relationships within the context of PD, and the results highlight its promising performance in addressing this challenge.

Within the realm of skeleton-based action recognition, various studies have embraced diverse strategies to enhance the performance of recognition models. Some have incorporated attention mechanisms to emphasize discriminative joints within each frame, while others have employed a spatial–temporal graph convolutional network (ST-GCN) as the foundational framework [41]. An ST-GCN effectively captures both spatial and temporal information by introducing graph convolution for spatial features and conventional convolution for dynamic temporal information [42]. To further elevate performance, certain studies have proposed enhancements to the ST-GCN, such as cross-domain spatial residual layers and dense connection blocks. These innovations effectively handle spatial–temporal information and enhance feature robustness, respectively [43,44]. Additionally, another study introduced variable temporal dense blocks with varying kernel sizes to extract temporal features across different ranges [45].

In [46], the authors present a novel model, known as the crow-search-algorithm-based decision tree (CSADT), for the early diagnosis of PD. The proposed method was rigorously tested on four distinct PD datasets: meander, spiral, voice, and speech-Sakar. Key highlights of the CSADT model include data normalization, novel locations generated through the crow search algorithm, sub-feature selection using the sigmoid function, and decision tree analysis. The CSADT model achieved remarkable accuracy, with results indicating close to 100% accuracy and swift diagnosis, demonstrating its potential for early PD detection. The CSADT model achieved close to 100% accuracy in the diagnosis of Parkinson’s disease, making it a promising tool for early detection. The model’s success is attributed to its innovative approach, including data preprocessing, the crow search algorithm, and decision tree analysis. It outperformed other machine learning algorithms in terms of accuracy, precision, recall, and the combination measure F1. This innovation offers a reliable and rapid diagnosis of PD. In [47], ML and DL techniques were employed to identify blood-based biomarkers for Alzheimer’s and Parkinson’s diseases, with a specific focus on the promising performance of CNNs for biomarker identification and disease detection, which holds potential for early diagnosis and clinical trial screening.

In recent years, the field of neuroimage analysis has witnessed the development of numerous data mining techniques, with a particular surge in the popularity of DL models, attributed to their accomplishments in diverse computer vision applications [48]. An illustrative example is the work by Ktena et al. [49], which introduced a metric learning approach aimed at distinguishing between cases and controls in autism research. This innovative method involves the construction of a graph representing patients’ brain networks in regions of interest (ROIs) utilizing GCN. It leverages this graph to extract features from patients’ neuroimages.

### 4.2. Graph Attention Networks (GATs)

GATs, a subset of GNNs, integrate attention mechanisms to evaluate the significance of connections within a graph. They have proved highly advantageous for analyzing graph-structured data in the context of PD research, where intricate relationships among various data sources, such as patient-reported outcomes, imaging data, and EHRs, can be portrayed as a graph. Using GATs, each node within a graph receives an attention weight based on its associations with other nodes. This weight governs the node’s importance within the network, influencing the information flow between nodes. Consequently, GATs possess the ability to dynamically assess the importance of diverse connections within the graph and focus on critical information during DP.

A notable strength of GATs in PD research lies in their capacity to manage extensive and intricate graphs featuring numerous nodes and connections. Leveraging attention mechanisms to adaptively weigh connections, GATs effectively filter out extraneous or irrelevant data and prioritize essential information during DP. Recent advancements in the application of GATs for PD diagnosis have emerged, notably utilizing DL models for the analysis of imaging data and prognosis of disease progression. These approaches have exhibited promising outcomes, boasting high accuracy rates in PD detection and prognostication, which could significantly enhance patient outcomes and alleviate the burden of PD on healthcare systems.

Additionally, researchers have explored the utilization of GCNs in the concurrent analysis of structural and functional MRI data to classify autism. In one study (Arya et al. [50]), relational data between nodes were extracted from T1w structural metrics, while functional brain summaries were derived from fMRI data, and subsequently employed within a GCN model. Another study (Dsouza et al. [51]) introduced a multimodal GCN (M-GCN) framework for predicting phenotypic measures, amalgamating inputs from functional connectivity (FC) and subject-specific structural connectomes. Moreover, the GAT model has been explored for its potential interpretability in predicting phenotypic measures within a bipolar dataset (Yang et al. [52]), using the FC matrix as the graph and an anatomical and statistical FC feature set. These endeavors showcase the ongoing exploration of graph-based NN models to advance our understanding of neurological conditions like PD and autism while potentially enhancing patient care.

This study proposes a deep multi-modal fusion model (DMFM) based on GAT as the method for capturing spatial dependencies. GAT is harnessed to model these graphs, effectively incorporating spatial dependencies. Furthermore, a combination of global context information and the allocation of adjacent time importance is achieved by integrating convolutional long short-term memory (ConvLSTM) and an attention mechanism into a temporal attention mechanism (TAM) to model the spatiotemporal correlation [53]. Finally, a prediction module is employed for making the ultimate prediction.

### 4.3. Graph Recurrent Networks (GRNs)

Graph recurrent networks (GRNs) represent a subtype of GNNs that incorporate recurrent connections, facilitating the modeling of dynamic changes in graph-structured data. Within the context of PD research, where alterations in patient symptoms, imaging data, and EHRs unfold over time and can be represented as a dynamic graph, GRNs prove to be well-suited for processing such time-series data.

In GRNs, every node within a graph maintains connections with itself across multiple time steps, enabling the network to effectively capture the temporal evolution of the graph. This characteristic is particularly advantageous when dealing with time-series data in the PD domain, where shifts in patient symptoms and clinical indicators can be aptly characterized as dynamic graph structures [54].

A notable advantage of GRNs in the realm of PD research lies in their capacity to capture temporal dependencies among various data sources, encompassing patient-reported outcomes, imaging data, and EHRs. By effectively modeling the evolving nature of a graph over time, GRNs contribute to a more comprehensive understanding of disease progression, thereby assisting healthcare providers in gaining deeper insights into the underlying mechanisms of PD. Recent advancements in the deployment of GRNs for PD diagnosis involve the application of DL models to analyze time-series imaging data and predict disease progression. These endeavors have yielded promising outcomes, characterized by high accuracy rates in PD detection and prognosis, suggesting the potential to enhance patient outcomes and alleviate the burden of PD on healthcare systems. In summation, GRNs represent a robust tool for processing and analyzing time-series data within the scope of PD research. Their utility holds substantial promise for advancing our comprehension of this intricate condition and, consequently, for improving patient outcomes [37].

### 4.4. Graph Transformer Networks (GTNs)

Graph transformer networks (GTNs) represent a subtype of GNNs that have been infused with the transformer architecture, initially conceived for sequential data like natural language. In the realm of PD research, GTNs have found utility via the adaptation of this architecture to process graph-structured data, manifesting their applicability in diverse domains. Within GTNs, the preservation of the graph structure is achieved through the employment of graph attention mechanisms. These mechanisms empower the network to assign varying degrees of importance to different nodes within the graph, a crucial attribute when dealing with the intricacies of PD research. In this context, where distinct clinical markers and patient-reported outcomes may hold differing degrees of relevance in predicting disease progression, the ability of GTNs to weigh such importance proves especially valuable.

An inherent strength of GTNs in the domain of PD research lies in their proficiency in managing substantial and intricate graph structures. This capability is particularly pertinent when dealing with complex data sources like EHRs and imaging data. By doing so, GTNs effectively capture the intricate interconnections between various data elements, thus furnishing a more comprehensive perspective on disease progression and patient outcomes [54]. Recent strides made in applying GTNs to the diagnosis and treatment of PD have been noteworthy. These advances encompass the utilization of DL models for scrutinizing imaging data and forecasting disease progression. Impressively, these approaches have exhibited considerable potential, yielding commendable accuracy rates in the realms of PD detection and progression prediction. Such advances hold substantial promise in the realm of healthcare systems by potentially ameliorating patient outcomes and mitigating the healthcare burden induced by PD.

It is worth noting that these approaches encompass distinct optimization techniques, with the first relying on the Adam optimizer and the second opting for the L-BFGS optimizer [55]. However, due to the limitations of the baseline implementation, the use of the Adam optimizer becomes a pragmatic choice, albeit necessitating additional hyperparameter fine-tuning to yield optimal results. Despite this requirement for meticulous tuning, the outcomes achieved with the Adam optimizer surpass those achieved through fast neural style transfer [55]. This enhancement can be attributed to the intricate nature of the task involving the creation of a versatile transformer network proficient in accommodating both the style and content signals.

### 4.5. Graph Autoencoders (GAEs)

Graph autoencoders (GAEs) constitute a noteworthy facet of GNNs applied to the intricate realm of PD research. Operating on the autoencoder architecture, GAEs are adept at processing graph-structured data, an attribute that aligns with the multifaceted nature of PD studies. Autoencoders, by design, are NNs proficient in the art of reconstructing their input data. They achieve this by encoding the data into a lower-dimensional representation and subsequently decoding it to regain the original form. In the context of GAEs, this input takes the form of a graph, while the lower-dimensional counterpart materializes as a graph embedding. Within the domain of PD research, GAEs prove invaluable in deriving concise representations from intricate graph structures, such as EHRs and imaging data. This proficiency enables GAEs to capture the underlying relationships interconnecting diverse data sources, thereby furnishing a more comprehensive understanding of disease progression and patient outcomes. Additionally, GAEs serve as adept tools for dimensional reduction—an asset of considerable importance in PD research, where handling high-dimensional datasets, notably in the realm of imaging data, poses distinct challenges. By condensing data into lower dimensions, GAEs simplify the process of identifying latent patterns and relationships, which might have eluded detection within the original data representation.

Noteworthy strides have been made in employing GAEs for PD diagnosis and therapeutic interventions. These advancements encompass the utilization of DL models for dissecting imaging data and forecasting disease progression. Encouragingly, these approaches have yielded promising outcomes, harboring substantial potential for enhancing patient well-being and alleviating the healthcare burden associated with PD. In conclusion, GAEs emerge as a vital and adaptable tool within the PD research landscape. Their competence in managing graph-structured data, coupled with their ability to streamline complex datasets through dimensionality reduction, positions them as valuable assets in unraveling the complexities of PD.

Other authors have introduced DD-GCN, a novel method for predicting human splice-site (SL) events using GCNs. DD-GCN employs a two-pronged drop-out strategy, including coarse-grained node dropout and fine-grained edge dropout, to enhance gene embeddings for precise SL prediction [56]. Importantly, DD-GCN exclusively trains on established SL pairs without additional data from external gene sources. Furthermore, the authors present the SLMGAE model, designed for predicting protein–protein interactions (PPIs) by considering multiple perspectives within a protein graph. Using an autoencoder architecture, SLMGAE obtains low-dimensional graph representations for predictive purposes. Experiments confirm SLMGAE’s superior predictive performance compared to other GNN methods and matrix factorization techniques [57,58]. In [59], the authors introduced two innovative Hessian-based SSFS frameworks, denoted as Hessian–Laplacian-based SSFS frameworks using the generalized uncorrelated constraint (HLSFSGU). These frameworks employ mixed-norm (0 < *p* < 1) regularization for joint sparse feature selection, presenting a novel approach to selecting informative features. Both the HLSFSGU framework and GAEs represent innovative approaches in their respective domains. The HLSFSGU framework introduces a novel method for feature selection by combining Hessian and Laplacian matrices, while GAEs are known for their unique approach to graph-based autoencoding. In summary, GAEs are valuable for analyzing graph-structured data in PD research, offering potential benefits in understanding the disease and improving patient outcomes.

### 4.6. Graph Generative Networks (GGNs)

Graph generative networks (GGNs) represent a category of graph-based DL models designed to produce new graph instances resembling a given graph or a collection of graphs. In the context of PD, GGNs can be employed to create realistic depictions of PD symptom progression over time, leveraging available PD patient data. These generated graphs serve as a foundation for predicting future PD symptom developments or gaining insights into the fundamental biological processes driving the disease. By integrating graph-based representations and generative models, GGNs furnish a potent instrument for unraveling the intricate interplay between PD symptoms and the underlying disease pathology, as well as for crafting more efficacious and personalized PD treatments [60].

In the domain of medical image analysis, where labeled images are often limited in availability, augmentation plays a pivotal role, facilitated by the GGNs-based data augmentation technique. GGNs constitute a generative framework comprising two networks: a generator network responsible for crafting synthetic data and a discriminator network tasked with distinguishing real data from synthetic counterparts.

### 4.7. Graph Reinforcement Learning Networks (GRLNs)

Graph reinforcement learning networks (GRLNs) are graph-based deep learning models that utilize reinforcement learning algorithms to make decisions based on graph-structured inputs. In the context of PD, GRLNs are valuable for making personalized treatment decisions, including optimizing medication dosages, selecting appropriate physical therapy exercises, and predicting symptom progression. By combining graph-based representations with reinforcement learning, GRLNs improve treatment choices for PD patients, leading to better outcomes. They can also be trained on large datasets to capture complex data patterns and relationships, resulting in more accurate predictions and enhanced treatment strategies.

In [61], a deep reinforcement learning network was developed to predict brain tumor locations. Using 70 post-contrast T1-weighted 2D image slices from the BraTS brain tumor imaging database, a deep Q-network (DQN) was trained to demonstrate reinforcement learning’s practical application in radiology AI. In another instance [57], researchers devised a deep-reinforcement-learning-based method for medical image semantic segmentation. The goal was to reduce human involvement in extracting medical image masks, introducing an advanced version of the deep Q-learning architecture. Although this technique showed promise in selecting optimal masks during image segmentation, the authors noted potential room for improvement in the mask extraction stage in future research. Lastly, in [60], a reinforcement-learning-based recommendation system for antihypertensive medications was proposed for patients with hypertension and type 2 diabetes. This system aimed to enhance precision medicine by integrating electronic health data and machine learning, resulting in the development of a Q-learning model. Table 2 provides an overview of the employment of various GNN techniques across the cited related works. The table offers valuable insights into the adoption of GNNs in different studies, showcasing the diversity of techniques employed for various research objectives.

Table 3 was constructed to encompass a range of criteria meticulously chosen to provide an all-encompassing characterization of the 12 distinct algorithms and tools employed within the domain of GNNs. The “Availability” criterion indicates whether the respective algorithm or tool is readily accessible to the public and whether it can be employed without financial constraints. The “Data Volume” criterion indicates a pivotal role in gauging the capability of the algorithm or tool to manage substantial data volumes, thereby addressing its aptitude for handling big data. Equally significant is the “Data Variety” criterion, as it discerns the algorithm/tool’s versatility in accommodating various data types and facilitating the integration of heterogeneous data sources. The “Data Velocity” criterion is instrumental in elucidating whether the algorithm/tool is tailored to function seamlessly with streaming data or is designed for static DP. In the healthcare context, “data veracity” alludes to the precision, comprehensiveness, and dependability of healthcare data, spanning data originating from medical imaging, electronic health records, wearable devices, and other sources. Assuring the veracity of healthcare data is a critical imperative, bearing implications for informed decision-making in patient care, treatment strategies, and healthcare policy formulation. The absence of data veracity, characterized by inaccuracies, omissions, or unreliability, can culminate in erroneous diagnoses, ineffective therapeutic interventions, and suboptimal patient outcomes. Hence, the preservation of data veracity assumes paramount importance in the realm of healthcare data management and analytics. Lastly, the “Monitoring/Alerting” criterion indicates significance in appraising whether the algorithm/tool boasts the essential functionality to monitor data and promptly notify patients and healthcare practitioners of any pertinent issues.

Table 3 includes various algorithms, assessing factors like availability, scalability, data handling (volume, variety, velocity), and monitoring/alerting capabilities. For example, MV-GCN is open-source, scalable, and handles homogeneous static data, but lacks monitoring and has low velocity. ST-GCN is similar but with medium velocity. ROI-GCN is a proof-of-concept, scalable, with low velocity and no monitoring. MRI-GCN is open-source and scalable, with low velocity and no monitoring. Other algorithms share similar characteristics. Some excel with heterogeneous data (e.g., multimodal GCN), while others suit homogeneous data (e.g., GRNs for PD diagnosis). The “DMFM based on GATs” is scalable, handles heterogeneous data, and has medium velocity and veracity, but lacks monitoring/alerting. None of the listed algorithms offer monitoring/alerting functionality.

## 5. Discussing Open Issues and Challenges

While GNNs are promising in the advancement of PD research, there are various open issues and challenges to be tackled in the future. In this section, we aim to explore and discuss the issues/challenges identified in this survey.

**Data Quality and Quantity**: The quality and quantity of data available for PD research are pivotal factors influencing the effectiveness of GNNs. This encompasses diverse data sources, including patient-reported outcomes, imaging data, and EHRs. Ensuring data quality and consistency poses a substantial challenge, underlying the necessity for robust and standardized data collection protocols.

Data quality and quantity constitute paramount considerations in applying GNNs within the realm of PD research. The performance of GNN models hinges directly upon the caliber and volume of available data. PD research draws data from various sources, including electronic health records, wearable devices, and self-reported surveys. The imperative lies in maintaining data accuracy, consistency, and relevance to the specific research problem. Additionally, the volume of data holds significance in GNNs for PD, as a larger dataset often translates into enhanced model generalization and improved performance. Nonetheless, accumulating substantial data quantities can prove arduous, particularly in the healthcare domain, where ethical and privacy concerns loom large. Hence, striking an equilibrium between data quality and quantity emerges as a fundamental endeavor to effectively harness the potential of GNNs in PD research.

In summation, the utility of GNNs in PD research is heavily reliant on the caliber and volume of available data. Factors such as data accuracy, consistency, relevance, and ethical considerations within the healthcare domain collectively influence the success of the research. Achieving the right balance between data quality and quantity serves as a linchpin for attaining meaningful and precise outcomes in PD research employing GNNs.

**Model Selection and Validation**: GNNs are a powerful but intricate tool, and choosing the appropriate ontology model for a specific dataset and application can be challenging. Additionally, ensuring the reliability of GNN performance through robust and standardized validation procedures is crucial.

In the context of PD research, selecting and validating GNN models are pivotal for accurate and dependable results. Model selection aims to identify a model capable of capturing the inherent relationships within PD data, especially concerning graph structures and node interactions.

Once a GNN model is chosen, rigorous validation is essential to gauge its generalizability to new, unseen data. Various techniques like cross-validation, holdout validation, and bootstrapping serve this purpose. Cross-validation involves dividing the data into subsets for iterative model training and evaluation. Holdout validation partitions the data into training and validation sets, with the model trained on the former and evaluated on the latter. Bootstrapping, a resampling method, repeatedly samples from the original dataset for training and evaluation. In summary, GNNs offer substantial potential in PD research, but their complexity demands careful model selection and comprehensive validation. Established techniques such as cross-validation, holdout validation, and bootstrapping are instrumental in this process, ensuring the trustworthiness of research outcomes.

When evaluating a GNN model, it is imperative to assess both its accuracy and robustness. Accuracy is typically measured through common metrics like precision, recall, and the F1-score, which provide insights into the model’s performance. However, in addition to accuracy, it is crucial to evaluate the model’s robustness in handling missing or noisy data. Furthermore, assessing the model’s generalizability to new, unseen data can be achieved through techniques like transfer learning.

In summary, the selection and validation of GNN models in the PD research domain play a pivotal role in ensuring the accuracy and reliability of outcomes. These evaluations should focus on the model’s capacity to grasp the intricate relationships within PD data and its adaptability to novel, unobserved data. The performance assessment should encompass a blend of precision and reliability metrics.

**Integration with Electronic Healthcare Records**: EHRs are pivotal in PD research, containing essential patient data encompassing demographics, medical history, and treatment outcomes. Nevertheless, harmonizing these data with GNNs presents a challenge, underlining the necessity for standardized protocols and data integration tools to facilitate their effective utilization in PD research.

In the realm of PD research, the fusion of EHRs with GNNs holds profound significance for tailoring personalized treatment strategies and guidelines. This integration empowers the analysis of a patient’s comprehensive health dossier, encompassing demographic particulars, symptomatology, medication chronicles, and diagnostic findings. This amalgamation furnishes the groundwork for an integrated knowledge graph, capitalizing on GNN capabilities to scrutinize and fathom PD data.

For the seamless integration of EHRs with GNNs within the PD domain, multifaceted technical and non-technical considerations merit attention. Factors including data privacy and security take precedence as the collected data may encompass sensitive health details. Interoperability is equally pivotal as EHRs are frequently archived in disparate formats, often necessitating conversion and standardization for GNN compatibility.

Moreover, ensuring the quality and quantity of collected EHR data is imperative to underpin the development of effective GNN models. Equally pivotal are the steps of model selection and validation in the amalgamation of EHRs with GNNs, as the chosen model significantly influences result accuracy and reliability.

In conclusion, the integration of EHRs with GNNs within the PD domain harbors substantial potential for advancing our comprehension of PD and facilitating the formulation of tailored treatment strategies and guidelines for PD patients. Nevertheless, it is imperative to confront technical and non-technical obstacles, encompassing data privacy, security, interoperability, data quality and quantity, as well as model selection and validation, to realize a successful integration.

**Privacy and Confidentiality**: The utilization of GNNs in PD research introduces paramount concerns regarding privacy and confidentiality, primarily due to the analysis of sensitive patient data. Safeguarding the privacy and confidentiality of these data is of utmost importance, necessitating the implementation of robust privacy and security protocols, as well as comprehensive data protection measures. Within the realm of PD research, the application of GNNs gives rise to substantial privacy and confidentiality considerations. This stems from the acquisition, retention, and processing of medically sensitive information pertaining to patients. Inadequate handling of such data could potentially result in security breaches, unauthorized access, and identity theft. To effectively mitigate these concerns, it is imperative to establish stringent privacy and security measures that ensure the safeguarding of patients’ personal information. This can be accomplished through the adoption of encryption techniques, access control mechanisms, and secure data storage practices. Additionally, adherence to pertinent data protection regulations, such as the General Data Protection Regulation (GDPR) [62], Health Insurance Portability and Accountability Act (HIPAA) [63], and the Personal Information Protection and Electronic Documents Act (PIPEDA) [64], is essential. Furthermore, the collection of medical information should only proceed with informed consent from patients, with data utilization strictly confined to the intended purpose. To uphold the confidentiality and privacy of PD patients’ information, it is advisable to implement a multifaceted approach encompassing both technical and organizational measures.

In summary, the incorporation of GNNs in PD research introduces a spectrum of complexities and unresolved matters that warrant further exploration. The paramount importance of data quality and quantity cannot be overstated, as it fundamentally underpins the efficacy of GNN models. Striking the right equilibrium between these factors holds significant implications for the attainment of precise and substantive outcomes. Furthermore, the meticulous selection and validation of models are pivotal steps in ensuring the resilience and broad applicability of GNN models. It is advisable to employ a fusion of accuracy and robustness metrics when assessing a model’s performance.

The potential integration of EHRs offers a promising avenue for harnessing substantial clinical data resources in PD research. However, it is imperative to acknowledge and address the pertinent concerns of privacy and confidentiality. Upholding responsible data handling practices is indispensable for cultivating trust and upholding ethical standards within healthcare research. In conclusion, the effective implementation of GNNs in PD research hinges on the resolution of these ongoing issues and challenges. Such endeavors are instrumental in advancing our capacity to devise more efficacious treatments for individuals afflicted by PD.

## 6. Proposed Approach

The proposed approach designed for PD employing GNNs primarily aims at the extraction of valuable insights to facilitate PD monitoring and alert systems. This approach emphasizes the integration of heterogeneous and disparate data from external sources, such as sensor data collected from wearables and EHRs, into a GNN-based framework.

The initial step of this proposed approach encompasses data retrieval and pre-processing, including the collection of raw data from external sources. In our experimental data collection, a Samsung Galaxy watch (46 mm SM-R800) was utilized, in conjunction with a custom application developed on the Tizen 4.0 OS and deployed on the watch [30]. The sensor data collected from the application on the watch undergo a series of preparatory steps to render them suitable for subsequent analysis. This preparatory step encompasses tasks such as data standardization, normalization, and the removal of superfluous or irrelevant information. This entails initial data standardization to ensure uniformity, normalization to bring data within a consistent scale, and the application of noise reduction techniques to eliminate any outliers or irrelevant information. Following the ontology-based semantic annotation and integration of the available data, the resulting knowledge graph serves as the foundation for the creation of the ultimate GNN model tailored for PD. This final GNN model comprises several integral components, including a GCN, a GAT, and a GRN.

These interconnected networks collaborate to systematically analyze the data and derive meaningful insights to bolster PD research and facilitate the creation of personalized treatment strategies. It is essential to underline that the proposed architectural framework must also effectively address privacy and confidentiality concerns, given that the data being collected and analyzed may encompass sensitive information. Implementing safeguards such as encryption and secure data storage becomes imperative to uphold patient privacy and maintain the confidentiality of their information.

As previously mentioned, the current method of monitoring and signaling medication dosing in PD relies on fixed-time alerts scheduled through smart devices, using DDIs entered by either the patient or their healthcare provider. However, this approach lacks real-time integration of critical health data, such as immediate symptoms or the patient’s condition, relying solely on patient actions. This limitation can lead to deviations from the prescribed treatment plan, resulting in suboptimal treatment adherence and effectiveness. To address this challenge and ensure the security and privacy of collected data, our proposed solution involves the development of an innovative edge-device-based application with robust data protection mechanisms. This system operates by continuously collecting and analyzing the personal health data of PD patients in real-time or near-real-time while adhering to stringent data security standards and patient privacy regulations. Utilizing a combination of symptom analysis, including factors such as bradykinesia and tremor, our approach accurately identifies instances of missed medication doses. By scrutinizing these subtle events, the application promptly detects deviations from the treatment plan and promptly alerts the patient. Our solution is designed to prioritize data security and patient privacy, incorporating encryption, access controls, and compliance with data protection regulations. This approach ensures that sensitive health data are handled with the utmost care and security.

In conclusion, the proposed architecture for PD monitoring and alerting, based on the utilization of GNNs, constitutes a comprehensive and systematic approach that not only harnesses the capabilities of GNNs to scrutinize and comprehend PD-related data but also places a strong emphasis on data security and privacy. Through the amalgamation of data from diverse sources and stringent data protection measures, this framework has the potential to usher in transformative changes in PD research and provide invaluable support for the formulation of personalized treatment regimens and guidelines tailored to PD patients while safeguarding the confidentiality of sensitive patient information.

**Figure 3:**
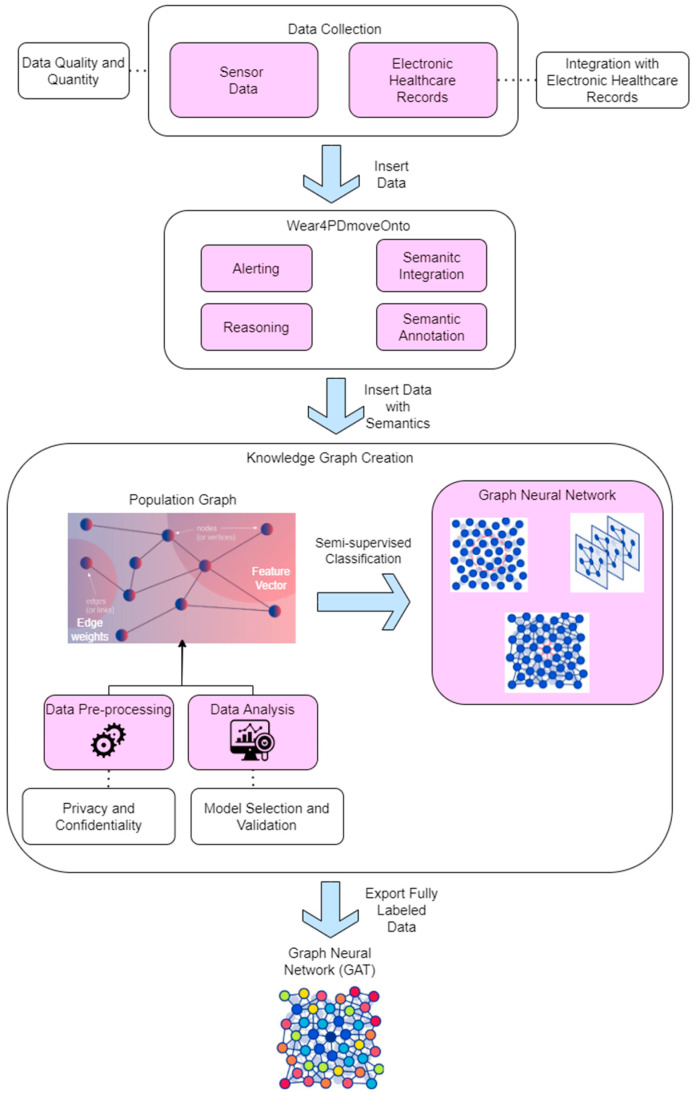
Proposed framework for creating GNNs for PD. New steps/tasks are indicated with no-colored rectangles linked to colored ones via dotted lines.

To harness the full potential of GNNs in PD research, there is a need for the adoption of a comprehensive and cohesive approach to GNN construction and utilization. The proposed approach is underpinned by a series of structured steps, as illustrated in Figure 3.

1. **Data collection**: The initial step involves the acquisition of data from diverse origins, encompassing wearable devices and individual healthcare documentation. The collected data encompass a range of information, including patient-reported outcomes, imaging records, and electronic healthcare documentation, among other sources.
2. **Data pre-processing**: The collected data necessitate preliminary processing and conversion into a knowledge graph. This process entails the retrieval of pertinent data from various sources, as well as the cleaning and standardization of the data to achieve a uniform format, ultimately resulting in their incorporation into a knowledge graph.
3. **Data analysis**: Subsequently, the knowledge graph undergoes a thorough analysis employing data analysis techniques to rectify interconnected data. This process may encompass the utilization of graph theory principles and ML algorithms to discern patterns and associations within the data, thereby providing valuable insights into the fundamental mechanisms of PD.
4. **Knowledge graph creation**: The knowledge graph is subsequently leveraged to construct a comprehensive representation tailored for healthcare professionals and policymakers. This process may encompass the application of graph visualization techniques to present the data in an intuitive and comprehensible manner, facilitating the creation of personalized treatment strategies and guidelines for individuals affected by PD.

An innovative approach to enhancing the construction of GNNs in PD research involves the development of a comprehensive system that integrates diverse data sources, including sensor data, wearable technology data, and EHRs. This system would leverage advanced algorithms and DP techniques to extract and amalgamate information from these various sources, ensuring the resulting dataset’s accuracy, consistency, and relevance. Furthermore, it would incorporate robust strategies to safeguard data privacy and confidentiality while facilitating their availability for research and analysis purposes. This innovative solution, by improving both the quality and quantity of data accessible for GNNs, holds the potential to yield more precise and dependable outcomes in PD research and contributes to the advancement of more efficacious treatments.

One promising strategy for enhancing the integration of EHRs with GNNs involves the establishment of a standardized data structure that is universally compatible with various EHR systems. This endeavor may entail the creation of a common data model that standardizes the representation of pertinent data fields and their relationships, streamlining the processes of data aggregation and analysis. Furthermore, the development of dedicated ML algorithms tailored for EHR data could address challenges such as missing or inconsistent data, potentially elevating the quality and quantity of data accessible for GNN analysis. These advancements have the potential to contribute to more precise and reliable outcomes in PD research.

Within the domain of knowledge graph creation, a potential avenue for innovation, aimed at addressing privacy and confidentiality concerns, involves the formulation of a privacy-preserving pre-processing technique tailored for GNNs. This technique could adopt a differential privacy framework, which introduces controlled noise into the data before they are employed for model training. This approach serves to safeguard the privacy of sensitive data while still enabling GNNs to derive insights from the pre-processed data. Additionally, the pre-processing step may encompass data anonymization procedures, such as hashing or tokenization, further bolstering the protection of sensitive information. In summation, the development of a privacy-preserving pre-processing technique holds the promise of ensuring the responsible and ethical utilization of GNNs within the healthcare context.

Furthermore, the application of automated ML (AutoML) techniques holds the potential to bring about a transformation in the domain of model selection and validation within GNNs. This transformation arises from the automation of the processes involved in choosing the most suitable model architecture and hyperparameters. Such automation offers the significant advantage of substantial reductions in time and resources that would otherwise be required for manual tuning. Through the utilization of AutoML, researchers gain the capability to efficiently evaluate numerous models and hyperparameters, ultimately selecting the one that demonstrates superior performance for the specific task at hand. Moreover, AutoML has the potential to mitigate the risk of overfitting by automatically implementing essential measures, including cross-validation and regularization, thereby ensuring the model’s capacity for robust generalization to new and unseen data sources.

To enhance the precision of characterizing and categorizing patient data associated with PD, a comprehensive approach will be employed. This approach entails the collection and integration of data from both sensors and electronic health records (EHRs) into an ontology structure. This central ontology will be augmented with inputs and connections from pertinent supplementary ontologies. The primary objective is to derive a knowledge graph that effectively represents the intricate relationships among various variables and patterns of symptoms associated with PD.

Following the extraction of the knowledge graph, multiple stages of data analysis will be systematically applied. The overarching aim is to derive a GNN capable of more accurately classifying and categorizing patient data. This GNN will harness the information encapsulated within the knowledge graph to uncover previously undisclosed interdependencies among diverse variables. This innovative approach is anticipated to substantially enhance accuracy in detecting underlying patterns and trends pertaining to PD.

The process involved in the development of a GNN comprises several essential stages, each contributing to the refinement of the model. These stages encompass data pre-processing, feature extraction, network architecture design, and training and testing. Data pre-processing entails the meticulous cleaning and preparation of data, ensuring its suitability for subsequent analysis. Feature extraction focuses on the identification of the most pertinent features for analysis. Network architecture design entails the specification of the GNN’s structure, encompassing factors like the number of layers and nodes. Subsequently, the training and testing phases involve the propagation of data through the GNN, necessitating the adjustment of network weights and biases to enhance accuracy.

The culmination of this rigorous process yields a GNN with heightened accuracy in the characterization and categorization of patient data associated with PD. This heightened accuracy holds the potential to significantly enhance the management of PD symptoms and ultimately lead to improved patient outcomes. In summary, the proposed approach to GNNs in PD research revolves around the creation of an all-encompassing and integrated knowledge graph. This knowledge graph harnesses the capabilities of GNNs to scrutinize and comprehend PD-related data. Through the constructive collaboration of data analysis and knowledge graph construction, this approach has the potential to catalyze innovations in PD research and facilitate the development of individualized treatment strategies and guidelines for PD patients.

## 7. Conclusions

GNNs have demonstrated substantial promise in the realm of PD research, offering novel insights and methodologies for comprehending this intricate medical condition. This survey has offered a comprehensive examination of the current state of the art in applying GNNs to PD research. It has encompassed a wide array of data sources, pre-processing techniques, and data analysis methodologies employed to delve deeper into the intricate mechanisms underlying PD. Despite the challenges and ongoing issues in this domain, our proposed GNN approach to PD monitoring and alerting has the potential to initiate a substantial transformation in PD research. This approach is poised to drive substantial progress, facilitating the development of highly tailored treatment strategies, and ultimately enhancing the quality of life for individuals grappling with PD. The primary aim of this research endeavor is to leverage the computational capabilities inherent in GNNs for a comprehensive analysis and understanding of PD-related data.

Looking ahead, our future research endeavors will seek to further elucidate and expand the horizons of GNN applications in the realm of PD. Specifically, we intend to explore several specific research directions, including:

- **Semantic data integration**: We will delve deeper into the effective integration of diverse PD-related data sources using ontologies, including wearable sensor data and EHRs. This involves optimizing data processing techniques and ensuring semantic data interoperability. Efficient scalable reasoning strategies and rules will be also examined.
- **Advanced algorithm development**: We will concentrate on the development of advanced GNN algorithms tailored to the unique requirements of PD research, emphasizing volume, variety, velocity, and veracity of data. This entails the creation of novel approaches for modeling intricate relationships within the data.
- **Validation and clinical implementation**: We will embark on efforts to validate the practicality of our GNN-based approach in clinical settings. Our research will involve collaborating with healthcare practitioners and policymakers to gauge the real-world impact of our methodology on PD diagnosis and treatment.
- **Ethical and privacy considerations**: As the usage of personal health data is central to our approach, we will thoroughly explore the ethical and privacy concerns surrounding the utilization of sensitive patient information. We intend to establish robust ethical guidelines for the responsible use of such data.

These future research directions are expected to guide our ongoing work, ensuring that the paradigm of GNNs in PD research is realized in practical applications. Our commitment to advancing our understanding of this complex medical condition and enhancing the care and outcomes for PD patients remains unwavering.

## Figures and Tables

**Table 1:** Utilized technologies in PD research, as presented in related work.

References	Machine Learning	Deep Learning	Neural Networks	Knowledge Graphs	GNNs	Data Preparation	EDA	Data Visualization
[12]	√							
[13]		√						
[14]							√	
[15]								
[16]						√		
[17]	√	√						
[18]	√	√						
[19]	√			√				
[20]						√		
[21]						√		
[22]						√		
[23]							√	
[24]							√	
[25]							√	
[26]					√			
[27]					√			
[28]				√				
[29]					√			
[32]								√
[33]	√	√						
[34]								√
[35]	√				√			
[36]	√				√			
[37]		√			√			
[38]		√						
[39]			√		√			

**Table 2:** Utilization of GNN techniques in the related work.

References	GCNs	GATs	GRNs	GTNs	GAEs	GGNs	GRLNs
[40,41,42,43,44,45,46,47,48,49]	√						
[50,51,52,53]		√					
[37,54]			√				
[55]				√			
[56,57,58,59]					√		
[60]						√	
[57,60,61]							√

**Table 3:** Evaluation of related works with specific criteria.

Algorithms/ Criteria	Availability	Data Volume	Data Variety	Data Velocity	Data Veracity	Monitoring /Alerting
Multi-view graph convolutional network MV-GCN [40]	OS	Scalable	Homogeneous	Static	Low	No
Spatial–temporal graph convolutional network (ST-GCN) [41]	OS	Scalable	Homogeneous	Static	Medium	No
Regions of interest using GCN (ROI-GCN) [42]	PoC	Scalable	Homogeneous	Static	Low	No
Crow search algorithm and decision tree (CSADT) for early PD diagnosis [46]	OS	Scalable	Homogeneous	Static	Low	No
Convolutional neural network (CNN) for Alzheimer’s (AD) and Parkinson’s disease (PD) [47]	OS	Scalable	Homogeneous	Static	Low	No
MRI-GCN [50]	OS	Scalable	Homogeneous	Static	Low	No
Multimodal GCN [51]	FoS	Scalable	Heterogeneous	Static	Low	No
Deep multi-modal fusion Model (DMFM) based on graph attention networks (GATs) [53]	FoS	Scalable	Heterogeneous	Static	Medium	No
GRNs for PD diagnosis [54]	PoC	Scalable	Homogeneous	Static	Low	No
L-BFGS hyperparameter tuning [55]	OS	Scalable	Homogeneous	Static	Low	No
DD-GCN for human splice-site [56]	OS	Scalable	Homogeneous	Static	Low	No
Multi-view graph autoencoder [57]	FoS	Scalable	Heterogeneous	Static	Low	No
Hessian–Laplacian-based SSFS framework [59]	PoC	Scalable	Homogeneous	Static	Low	No
Medical image analysis GGNs [60]	OS	Scalable	Homogeneous	Static	Low	No
Deep Q-network (DQN) from BraTS brain tumor imaging [61]	OS	Scalable	Homogeneous	Static	Medium	No

## Data Availability

Not applicable.

## Abbreviations

PD	Parkinson’s disease
ML	Machine learning
DL	Deep learning
NNs	Neural networks
GNNs	Graph neural networks
MRI	Magnetic resonance imaging
AI	Artificial intelligence
PSL	Probabilistic soft logic
ILP	Inductive logic programming
EDA	Exploratory data analysis
DDIs	Drug–drug interactions
GCN	Graph convolutional networks
MV-GCN	Multi-view graph convolutional network
FC	Functional connectivity
ROI	Regions of interest
DTI	Diffusion tensor imaging
DMFM	Deep multi-modal fusion model
PPMI	Parkinson’s Progression Markers Initiative
ST-GCN	Spatial–temporal graph convolutional network
GAT	Graph attention networks
M-GCN	Multimodal graph convolutional network
EHR	Electronic healthcare records
MF-GCN	Multimodal fusion graph convolutional network
ConvLSTM	Convolutional long short-term memory
TAM	Temporal attention mechanism
GRN	Graph recurrent networks
GTN	Graph transformer networks
L-BFGS	Limited-memory Broyden–Fletcher–Goldfarb–Shanno
GAE	Graph autoencoders
DD-GCN	Dynamic directed graph convolutional network
SLMGAE	Structure-preserved low-rank graph autoencoders
HLSFSGU	Hessian–Laplacian-based SSFS (space-saliency fingerprint selection) framework using generalized uncorrelated constraint
GGN	Graph generative networks
GRLN	Graph reinforcement learning networks
DQN	Deep Q-network
GDPR	General Data Protection Regulation
HIPAA	Health Insurance Portability and Accountability Act
PIPEDA	Personal Information Protection and Electronic Documents Act
